# Physiological Effects of Far-Infrared-Emitting Garments on Sleep, Thermoregulation, and Autonomic Function Assessed Using Wearable Sensors

**DOI:** 10.3390/s26020550

**Published:** 2026-01-14

**Authors:** Masaki Nishida, Taku Nishii, Shutaro Suyama, Sumi Youn

**Affiliations:** 1 Faculty of Sport Sciences, Waseda University, 2-579-15 Mikajima, Tokorozawa 359-1192, Japan; taku241@toki.waseda.jp (T.N.); shutaro.suyama@ruri.waseda.jp (S.S.); s2565174@u.tsukuba.ac.jp (S.Y.); 2 Sleep Research Institute, Waseda University, 513 Waseda-Tsurumakicho, Shinjuku, Tokyo 162-0041, Japan

**Keywords:** far-infrared radiation, wearable sensors, thermoregulation, sleep architecture, sweating rate, heart rate variability, textile technology

## Abstract

**Highlights:**

**What are the main findings?**

- A multimodal wearable sensing system enabled simultaneous evaluation of sleep architecture, thermoregulation, and autonomic activity under far-infrared (FIR) and control garment conditions.
- FIR garments modestly enhanced nocturnal heat dissipation, reflected by lower tympanic temperature and reduced sweating during the mid-sleep period.
- The proportion of rapid eye movement (REM) sleep increased without changes in total sleep time, indicating a redistribution of sleep stages under altered thermal conditions.

**What is the implication of the main findings?**

- These findings support the physiological feasibility of assessing functional sleepwear using integrated measures of thermal regulation and autonomic activity.
- Multimodal wearable sensing provides a useful framework for objectively evaluating subtle sleep-related effects of textile-based interventions.

**Abstract:**

Far-infrared (FIR)-emitting textiles are increasingly used in sleepwear; however, their influence on sleep physiology has not been comprehensively evaluated with multi-modal wearable sensing. This randomized, double-blind, placebo-controlled crossover study examined whether FIR-emitting garments modulate nocturnal thermoregulation, autonomic activity, and sleep architecture. Fifteen healthy young men completed two overnight laboratory sleep sessions wearing either FIR-emitting garments or visually matched polyester controls. Tympanic membrane temperature (TMT), sweating rate, skin temperature, and humidity were continuously monitored using wearable sensors, and sleep stages and heart rate variability (HRV) were assessed using validated portable systems. Compared with control garments, FIR garments produced consistently lower TMT across the night (*p* = 0.004) and reduced mid-sleep sweating (condition × time interaction: *p* = 0.026). The proportion of rapid eye movement (REM) sleep was higher in the FIR condition (22.2% ± 6.5% vs. 18.6% ± 6.5%, *p* = 0.027), despite no changes in total sleep time or sleep efficiency. A transient increase in low-frequency power during early sleep (*p* = 0.027) suggested baroreflex-related thermal adjustments without sympathetic activation. These findings indicate that FIR-emitting garments facilitate mild nocturnal heat dissipation and support REM expression, demonstrating their potential as a passive intervention to improve sleep-related thermal environments.

## 1. Introduction

Sleep is strongly influenced by the surrounding thermal environment and associated heat-transfer mechanisms, which continuously interact with autonomic and circadian processes [1,2]. Optimizing the microclimate close to the skin—including temperature and humidity—can improve sleep onset, sleep stage stability, and the nocturnal decline in core body temperature (CBT) [3,4,5]. Prior research has predominantly modulated the sleep environment through bedding characteristics, mattress structure, or ambient climate control, demonstrating their roles in sleep depth and thermoregulatory efficiency [5,6]. In contrast, sleepwear, despite being in direct contact with the skin and contributing substantially to conductive and evaporative heat transfer, has received little objective evaluation in the context of sleep physiology [7,8,9].

Fiber composition and textile structure influence thermal insulation, moisture wicking, and evaporative heat loss [8,9]. Furthermore, advances in stretchable and piezoelectric textile-based sensors have demonstrated that wearable fabrics can integrate mechanical strain–sensor functionalities, potentially enabling future multi-modal monitoring beyond thermal indices [10,11]. However, few studies have combined sleepwear interventions with continuous monitoring of thermophysiological variables such as deep-body temperature fluctuations, sweating rates, and autonomic dynamics across sleep cycles. Given the rapid temporal variation in these processes throughout NREM–REM transitions [12], subjective or intermittent measurements are insufficient for capturing textile–body interactions during sleep. Advances in multi-modal wearable sensors now allow high-resolution recording of these physiological parameters under controlled laboratory conditions [13,14,15,16].

Far-infrared (FIR)-emitting textiles incorporate ceramic nanoparticles that enhance emissivity in the 5–20 μm band, corresponding to the human body’s peak thermal radiation spectrum [17]. FIR radiation may promote peripheral vasodilation and radiative heat dissipation [18,19,20], suggesting potential relevance for sleep-related thermoregulation. Preliminary studies using FIR blankets or pajamas have reported subjective improvements or changes in selected sleep stages [21,22,23]; however, their findings were not supported by multi-parametric physiological measurements, leaving the underlying mechanisms unclear.

FIR-emitting garments exert thermophysiological effects that may influence sleep, including enhanced surface heat dissipation through vasodilation and radiative exchange [19,20,21,22]. These processes contribute to the reduction in core body temperature required for sleep initiation and maintenance and may therefore benefit nocturnal thermoregulation [4,5]. Although limited pilot studies have reported subjective improvements and modest changes in sleep staging when using FIR-based sleep products [21,22,23], they did not incorporate continuous, multimodal physiological monitoring, leaving essential questions unanswered.

In particular, REM sleep regulation is highly sensitive to thermal state [24,25,26], and FIR-induced alterations in heat-release dynamics may influence the expression or stability of REM episodes. Similarly, sweating responses vary across the night depending on thermoeffector activation, but no prior study has characterized time-specific changes in evaporative cooling when FIR garments are worn during sleep. Furthermore, the link between nocturnal heat dissipation and autonomic adjustments remains unclear.

To address these gaps, the present study employed validated wearable sensors to continuously monitor tympanic temperature, sweating rate, skin temperature, humidity, electroencephalography, and heart rate variability during sleep under standardized laboratory conditions.

We therefore hypothesized that FIR-emitting garments would:

(1) Enhance nocturnal heat dissipation, reflected by lower tympanic temperature and reduced sweating;
(2) Modify sleep structure, particularly facilitating the expression of REM sleep;
(3) Induce transient autonomic adjustments during early sleep without sympathetic activation.

Through this integrated approach, this study aims to provide the first comprehensive physiological characterization of FIR sleepwear during human sleep, clarifying how wearable textile technologies may influence thermoregulation, sleep stages, and autonomic dynamics.

## 2. Materials and Methods

### 2.1. Study Design

The study employed a randomized, double-blind, placebo-controlled crossover design to assess whether FIR-emitting garments modulate thermoregulatory and sleep physiology. Each participant completed two overnight sessions (FIR vs. control), separated by a ≥3-day washout period. Both participants and investigators responsible for data collection and scoring were blinded to garment allocation.

### 2.2. Participants

Fifteen healthy male university students (20–21 years) without a history of sleep disorders, chronic illness, or medication use participated. All maintained regular sleep–wake schedules for ≥1 week before laboratory visits and avoided caffeine and alcohol for 24 h. The required sample size was determined a priori using G*Power version 3.1.9.7 (η^2^ = 0.25, α = 0.05, 1 − β = 0.80), indicating *n* = 15 as sufficient [27]. Ethical approval was obtained from the Waseda University Academic Research Ethical Review Committee (Approval No. 2022-037), and all participants provided written informed consent.

### 2.3. FIR and Control Garments

The FIR garment consisted of polyester fibers embedded with ceramic nanoparticles emitting in the 5–20 μm band [18,19,20]. The control garment was visually identical and matched for fabric weight, thickness, and knit structure, differing only in the absence of FIR-emitting components.

Manufacturer-provided spectroscopic testing confirmed FIR emissive properties. Fiber composition details were not disclosed due to proprietary restrictions but are not required for physiological evaluation.

### 2.4. Procedures

Participants arrived at 20:00, instrumentation was applied, and the assigned garment was worn throughout the recording. The sleep opportunity was fixed from 23:00 to 07:00 in a temperature- and humidity-controlled chamber (25.0 ± 0.3 °C; 55 ± 3% RH). Light was maintained at <10 lux and noise < 30 dB. Upon waking, subjective sleep quality was assessed using the Oguri–Shirakawa–Azumi Sleep Inventory [28].

### 2.5. Physiological Signal Acquisitions

Sleep staging: EEG-based staging was performed using a validated wearable forehead-and-neck device (Insomnograf^®^ K2; S’UIMIN Inc., Tokyo, Japan) with >95% agreement with PSG [14]. Scoring followed AASM standards [29]. Thermoregulation: Tympanic membrane temperature, sweating rate, skin temperature, and skin humidity were continuously recorded (BL100; Technonext, Chiba, Japan) at 1 Hz [15].

Autonomic activity: HRV was recorded using Actiheart 5 (128 Hz; CamNtech Ltd., Cambridge, UK). Artifact correction applied a ±20% R-R filter; frequency-domain metrics (LF, HF) were log-transformed [16]. Device tolerance: Both EEG and ECG systems are validated for overnight monitoring, minimizing discomfort and known to exert negligible effects on sleep architecture [14,16]. Sensor placement and measurement configuration are shown in Supplementary Figure S1.

### 2.6. Data Segmentation and Analysis

Signals were aligned relative to sleep onset to evaluate time-dependent responses across sleep cycles. Statistical analyses used two-tailed paired *t*-tests for nightly averages and two-way repeated-measures ANOVA for time-course variables (Condition × Time), with Bonferroni-adjusted post hoc tests applied when appropriate. Effect sizes are presented as partial η^2^. Significance was set at *p* < 0.05 (SPSS v29.0, IBM, Armonk, NY, USA).

## 3. Results

### 3.1. Subjective Sleep

Subjective sleep quality ratings did not differ significantly between the FIR and control conditions (*p* > 0.10), indicating that participants were unaware of any perceptible differences in comfort or sleep experience during single-night use (Table 1).

### 3.2. Sleep Variables

Sleep efficiency and total sleep time did not differ between conditions (*p* > 0.10), confirming that the garments did not influence overall sleep duration or continuity. In contrast, the proportion of REM sleep was significantly higher in the FIR condition compared with control (22.2% ± 6.5% vs. 18.6% ± 6.5%, *p* = 0.027), despite unchanged NREM parameters (Table 2). These findings indicate a stage-specific effect, suggesting that FIR garments may facilitate REM sleep stability without altering sleep initiation or deep sleep expression.

### 3.3. Thermoregulation

**Figure 1:**
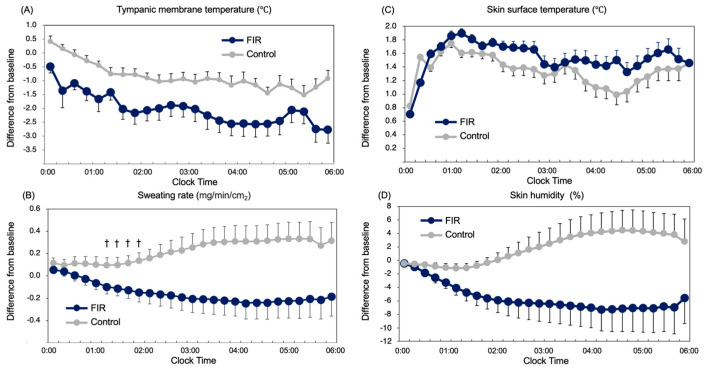
Comparisons of differences from baseline in measured variables over actual time clock. (**A**) Tympanic membrane temperature (TMT): A two-way repeated measures ANOVA revealed a significant main effect of time (*F*(3.66, 32.90) = 30.42, *p* < 0.001). (**B**) Sweating rate: A significant interaction between time and garment condition was observed (*F*(1.32, 22.43) = 2.121, *p* = 0.039) and a significant main effect of garment condition was found (*F*(1, 17) = 4.824, *p* = 0.042). (**C**) Skin surface temperature: No significant interaction or main effects. (**D**) Skin humidity: No significant interaction or main effects. Data are expressed as the difference from baseline. Error bars indicate standard error. Blue indicates FIR; gray indicates control. † *p* < 0.05 (Bonferroni-adjusted).

Tympanic membrane temperature (TMT) was consistently lower in the FIR condition compared with control across the sleep period (main effect of condition: *p* = 0.004) (Figure 1A). The largest between-condition difference emerged during mid-sleep, coinciding with the physiological nadir of core temperature.

Sweating rate demonstrated a significant condition × time interaction (*p* = 0.026), with reduced sweating specifically during mid-sleep under the FIR condition (Figure 1B). No differences were observed during early or late sleep (*p* > 0.10).

**Figure 2:**
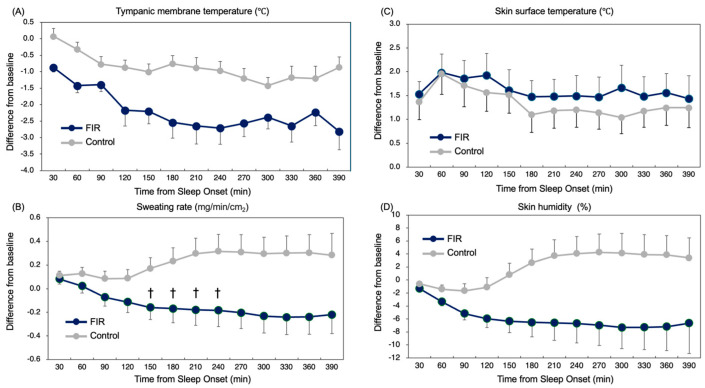
Comparisons of differences from baseline in measured variables after sleep onset. (**A**) Tympanic membrane temperature (TMT): A significant main effect of time was observed (*F*(4.23, 80.45) = 10.135, *p* = 0.002, η^2^ = 0.195), indicating a temporal decline in tympanic temperature. There was a significant main effect of condition (FIR vs. control) (*F*(1, 19) = 10.85, *p* = 0.004, η^2^ = 0.363), showing lower temperatures in the FIR condition. (**B**) Sweating rate: A significant interaction between time since sleep onset and garment condition was observed (*F*(1.71, 32.43) = 4.35, *p* = 0.026). The main effect of condition was also significant (*F*(1, 19) = 5.826, *p* = 0.026). Post hoc analyses revealed significant differences at 150 and 240 min after sleep onset. † *p* < 0.05 (Bonferroni-adjusted). (**C**) Skin surface temperature: No significant interaction or main effects. (**D**) Skin humidity: No significant interaction or main effects. Error bars indicate standard error. Blue indicates FIR; gray indicates control.

These results (Figure 1 and Figure 2) indicate that FIR garments enhanced nocturnal heat dissipation, reducing evaporative heat loss when thermoeffector demand is highest, while preserving normal thermoregulatory dynamics at other times of night.

### 3.4. Autonomic Activity

**Figure 3:**
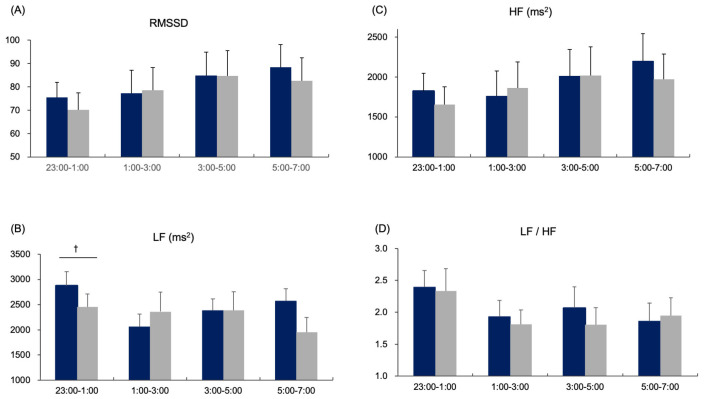
Changes in heart rate variability (HRV) indices across four time blocks during sleep under far-infrared (FIR) and control garment conditions. (**A**) RMSSD: No significant differences were observed between conditions across the night, indicating stable parasympathetic tone. (**B**) Low-frequency (LF) power: LF power was transiently higher in the FIR condition during the early sleep period compared with the control condition. (**C**) High-frequency (HF) power: HF power did not differ between conditions at any time point, further supporting preserved parasympathetic activity. (**D**) LF/HF ratio: The LF/HF ratio showed no consistent between-condition differences across the night. Data are presented as mean ± standard error. Blue bars indicate the FIR condition, and gray bars indicate the control condition. † *p* < 0.05 versus control (Bonferroni-adjusted).

Low-frequency power (LF) during early sleep was significantly higher in the FIR condition compared with control (*p* = 0.027), whereas high-frequency power (HF) and RMSSD did not differ between conditions (*p* > 0.10) (Figure 3).

These findings suggest a transient baroreflex-related adjustment associated with enhanced heat dissipation, without evidence of sympathetic activation or altered parasympathetic tone.

## 4. Discussion

This study investigated the thermoregulatory, autonomic, and sleep-related effects of FIR-emitting garments using multi-modal wearable sensing under controlled environmental conditions. The principal findings were that: (1) FIR garments produced consistently lower tympanic membrane temperature (TMT) across the night, (2) sweating rate was reduced during mid-sleep, (3) the proportion of REM sleep increased in the FIR condition despite unchanged total sleep time and sleep efficiency, and (4) autonomic responses were limited to a transient early-night increase in LF power without changes in HF or RMSSD. Together, these results indicate that FIR-emitting textiles can modestly alter the nocturnal thermal environment in a manner that is detectable with continuous physiological monitoring.

The reduction in TMT observed in the FIR condition is consistent with the proposed effects of FIR textiles on peripheral microcirculation and heat exchange [19,20]. FIR wavelengths in the 5–20 μm range penetrate superficial tissue layers and may promote vasodilation and enhanced radiative heat loss [18,19]. In the present study, continuous TMT monitoring revealed a persistent between-condition difference rather than a stage-specific or transient effect, suggesting that FIR garments facilitated mild but sustained nocturnal heat dissipation. This pattern aligns with prior work showing that small changes in peripheral heat loss can meaningfully influence nocturnal CBT dynamics and sleep regulation [4,5].

The time-specific reduction in sweating during mid-sleep further supports the interpretation that FIR garments improved the efficiency of nocturnal heat dissipation. Mid-sleep typically coincides with the nocturnal minimum of core temperature, at which point evaporative cooling becomes a dominant thermoeffector mechanism [4,5]. Under these conditions, the lower sweating rate in the FIR condition, despite equivalent ambient temperature and humidity, suggests that the garments reduced the physiological need for evaporative heat loss. In other words, FIR textiles appear to lower thermal load sufficiently that less sweating is required to maintain an appropriate body temperature. Although we matched FIR and control garments for color, fabric weight, thickness, and knit structure, we did not directly quantify textile thermal resistance or evaporative capacity. Differences in moisture transport or local heat flux may therefore also contribute to the observed sweating patterns. These findings highlight the importance of combining textile-level measurements with physiological monitoring in future work.

The increase in REM sleep percentage in the FIR condition may be related to the altered nocturnal thermal profile. Although absolute REM duration did not differ between conditions, the proportion of REM sleep increased, likely reflecting the stability of total sleep time across sessions. REM sleep is highly sensitive to thermoregulatory state [23,24], and its stability is facilitated by a mild decline in core temperature and reduced skin moisture. The lower TMT and humidity observed during the middle portion of the night likely contributed to an internal environment more conducive to REM expression [24,25]. REM expression depends on a state of thermoneutrality, in which active thermoregulatory defenses are minimized [26,30]. By lowering heat load, FIR garments may reduce the need for evaporative cooling and peripheral vasomotor adjustments, thereby facilitating REM initiation and maintenance. Because total sleep time did not change, this effect likely reflects a redistribution of sleep stages rather than an absolute extension of REM. Although the magnitude of change was modest, even small thermal adjustments can influence REM stability in individuals or environments sensitive to thermal disturbances. FIR textiles may thus offer a passive means of enhancing microclimate stability during sleep.

The observed increase in REM sleep proportion under the FIR condition may be explained by improvements in nocturnal thermal stability. REM sleep is known to emerge preferentially when thermoregulatory demand is reduced and vasomotor adjustments are minimized [24,25,30]. FIR-induced reductions in thermal load—reflected by lower TMT and reduced evaporative cooling demands—may therefore facilitate REM initiation and maintenance. Importantly, REM expression increased without changes in total sleep time or NREM architecture, indicating a redistribution of sleep stages rather than an extension of sleep duration. These findings align with evidence that even modest improvements in thermal comfort can support REM stability in environments susceptible to thermal disturbances [4,5].

The transient increase in LF power during early sleep may reflect baroreflex-mediated cardiovascular adjustments associated with enhanced heat dissipation [31], rather than sympathetic arousal or stress-related activation [32,33]. The absence of changes in HF and RMSSD further supports a stable parasympathetic tone and suggests that autonomic responses were physiologically adaptive and non-alerting. In addition, subjective sleep evaluations did not differ between conditions, indicating that FIR garments did not induce noticeable discomfort or disrupt perceived sleep quality. Collectively, the present results indicate that FIR garments subtly improve the thermal environment during sleep, with corresponding benefits to REM expression and transient autonomic modulation, while preserving overall sleep continuity and cardiovascular parasympathetic balance.

This study has several strengths, including its randomized double-blind crossover design, controlled environmental conditions, and the integration of multiple wearable sensing modalities. By synchronizing EEG, temperature, sweating, humidity, and HRV measurements, we were able to capture the dynamic interplay between thermal state, autonomic activity, and sleep architecture with high ecological validity. This sensor-based physiological characterization provides a more comprehensive framework for understanding textile–body interactions during sleep than has been available in prior FIR studies.

FIR-emitting garments may represent a practical, passive strategy for improving thermal comfort during sleep. Unlike ambient cooling or mattress-based interventions, sleepwear is directly coupled to the skin–textile interface, enabling continuous modulation of radiative and evaporative heat exchange without behavioral effort or additional energy consumption. Such features may be valuable in environments where thermoregulation is challenged, such as hot seasons, travel, athletic recovery, or housing conditions with limited climate control. However, the present findings are modest in magnitude and should be interpreted as physiological feasibility, rather than clinical efficacy.

This work demonstrates the advantages of integrating multi-modal wearable sensing with textile interventions to capture time-dependent thermophysiological responses during sleep. Continuous measures of TMT, sweating, and autonomic dynamics allowed us to identify stage-specific effects that would not be detectable with subjective assessments or single-parameter monitoring. This approach may facilitate evidence-based evaluation of smart or functional textiles, supporting rational design of sleep-related garments aimed at optimizing microclimate stability.

Several limitations warrant consideration. The sample consisted of healthy young men, and results may not generalize to women, older adults, or individuals with sleep or thermoregulatory impairments. The FIR and control garments differed in fiber composition, although they were matched for tactile properties and thickness; long-term comfort and durability were not assessed. Finally, we did not measure direct textile properties such as thermal resistance or emissivity in situ. Future studies should evaluate diverse populations, incorporate textile characterization, and examine whether FIR garments have clinically meaningful benefits under conditions of greater thermal strain or repeated use.

## 5. Conclusions

FIR-emitting garments modestly enhanced nocturnal heat dissipation, reflected by consistently lower tympanic temperature and reduced sweating during mid-sleep, and were associated with a small but significant increase in REM sleep, without changes in total sleep time or parasympathetic tone. These findings indicate that FIR sleepwear can subtly improve the thermal microclimate during sleep through passive radiative mechanisms.

Multi-modal wearable sensing enabled the first comprehensive physiological evaluation of FIR textiles during human sleep, revealing stage- and time-specific effects that would not be detectable with subjective measures alone.

While the physiological effects observed were modest and limited to healthy young men, this approach provides a basis for evidence-based development of functional sleepwear, particularly in contexts where thermoregulation is challenged. Further research is warranted to examine longer-term use and potential benefits in diverse populations.

## Figures and Tables

**Table 1:** Subjective sleep quality assessed using the Oguri–Shirakawa–Azumi Sleep Inventory.

Parameters	FIR	Control	t	*p*	d
Sleepiness on rising	49.6 (6.6)	48.4 (7.0)	0.715	0.486	0.169
Initiation and maintenance of sleep	39.5 (7.2)	38.8 (12.8)	0.207	0.839	0.066
Frequent dreaming	47.6 (9.5)	50.6 (9.9)	1.107	0.287	0.316
Feeling refreshed on waking	50.7 (6.7)	47.7 (8.3)	1.413	0.179	0.397
Sleep length	47.2 (8.1)	45.4 (11.8)	0.602	0.557	0.174

Abbreviations: FIR, Far-infrared. Values are presented as mean (standard deviation) for FIR and control conditions. Statistical comparisons were conducted using two-tailed paired *t*-tests.

**Table 2:** Comparison of objective sleep variables between FIR and control garment conditions.

	Variables	FIR	Control	t	*p*	d
Time in Bed	Total recording time (TRT) (min)	400.0 (6.7)	402.6 (6.4)	0.167	0.870	0.053
	Total sleep time (TST) (min)	366.7 (6.1)	381.0 (6.4)	0.708	0.490	0.253
	Sleep onset latency (min)	33.1 (0.6)	43.7 (0.7)	0.981	0.343	0.255
	Sleep efficiency (TST/TRT) (%)	84.5 (12.2)	84.7 (12.7)	0.053	0.959	0.019
	Wake after sleep onset (min)	36.4 (0.6)	27.7 (0.5)	0.479	0.639	0.185
Sleep stages (absolute time)	N1 (min)	31.7 (0.5)	35.3 (0.6)	0.715	0.486	0.230
	N2 (min)	178.3 (3.0)	201.0 (3.4)	1.798	0.094	0.604
	N3 (min)	73.1 (1.2)	72.6 (1.2)	0.041	0.968	0.013
	REM (min)	83.3 (1.4)	71.4 (1.2)	1.620	0.108	0.379
Sleep stages (relative time)	%N1 (% TRT)	9.0 (5.0)	9.4 (3.4)	0.282	0.782	0.084
	%N2 (% TRT)	48.6 (5.5)	53.0 (8.1)	1.631	0.125	0.636
	%N3 (% TRT)	20.1 (8.4)	18.9 (8.1)	0.506	0.620	0.143
	%REM (% TRT)	22.2 (6.5)	18.6 (6.5)	2.465	*0.027*	0.567

Abbreviations: FIR, Far-infrared; TRT, total recording time; TST, total sleep time; REM, rapid eye movement; N1–N3, non-REM sleep stages 1–3. Values are presented as mean (standard deviation). Statistical significance was evaluated using two-tailed paired *t*-tests.

## Data Availability

The dataset supporting this article is available at https://github.com/masakinishida/Sleepgarment (accessed on 1 November 2025).

## Supplementary Materials

The following supporting information can be downloaded at: https://www.mdpi.com/article/10.3390/s26020550/s1, Figure S1. Sensor placement and measurement configuration.
