# Supplementary material for: Physiological Effects of Far-Infrared-Emitting Garments on Sleep, Thermoregulation, and Autonomic Function Assessed Using Wearable Sensors
Source: Sensors (Basel). 2026 Jan 14;26(2):550. doi: 10.3390/s26020550 (PMC12845630; doi:10.3390/s26020550)
Supplement: Supplementary file 1 [file sensors-26-00550-s001.zip › sensors-4025824-supplementary.pdf]

Supplementary

Illustration of the placement of all physiological sensors used in the study. Tympanic membrane temperature (TMT) was measured using an in-ear thermistor probe positioned in the external auditory canal. Skin temperature and skin humidity sensors were attached to the chest and forearm using soft adhesive holders. Heart rate variability (HRV) was recorded with the Actiheart 5 module placed on the left chest. Sleep staging was assessed using the Insomnograf K2 wearable EEG device positioned on the forehead. The schematic reflects relative placement only and is not drawn to anatomical scale.

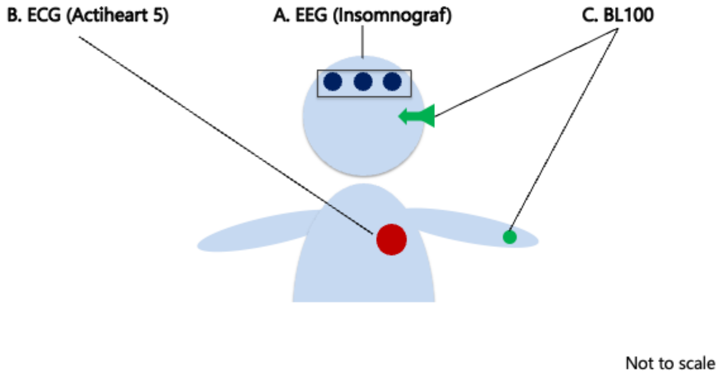

Figure S1. Sensor placement and measurement configuration.
